# RNAi and chemogenetic reporter co-regulation in primate striatal interneurons

**DOI:** 10.1038/s41434-021-00260-y

**Published:** 2021-05-19

**Authors:** Walter Lerchner, Abdullah A. Adil, Sekinat Mumuney, Wenliang Wang, Rossella Falcone, Janita Turchi, Barry J. Richmond

**Affiliations:** Laboratory of Neuropsychology, NIMH, NIH, Bethesda, MD USA

**Keywords:** Cellular neuroscience, Genetic vectors

## Abstract

Using genetic tools to study the functional roles of molecularly specified neuronal populations in the primate brain is challenging, primarily because of specificity and verification of virus-mediated targeting. Here, we report a lentivirus-based system that helps improve specificity and verification by (a) targeting a selected molecular mechanism, (b) in vivo reporting of expression, and (c) allowing the option to independently silence all regional neural activity. Specifically, we modulate cholinergic signaling of striatal interneurons by shRNAmir and pair it with hM4Di_CFP, a chemogenetic receptor that can function as an in vivo and in situ reporter. Quantitative analyses by visual and deep-learning assisted methods show an inverse linear relation between hM4Di_CFP and ChAT protein expression for several shRNAmir constructs. This approach successfully applies shRNAmir to modulating gene expression in the primate brain and shows that hM4Di_CFP can act as a readout for this modulation.

## Introduction

The use of modern genetic tools through germ line manipulation has led to a revolution in untangling the function of brain circuitry in small animals, especially flies and mice [1]. The application of genetic tools has moved more slowly in animals with larger brains, such as old-world monkeys [2, 3]. In these animals the generation time is so long that germ-line manipulation is impractical, so genetic material is usually delivered into neurons by local injections of nonreplicating viruses, such as lentivirus [4], adeno-associated-virus [5], or canine-adeno-virus 2 [6]. This approach has several limitations compared to germ line transmission. First, cell-type specific virus targeting is limited by a scarcity of cell-type specific promoters that can be packaged into viruses [7] and/or a lack of serotypes targeting individual cell populations [8]. Second, penetrance and expression often vary considerably across individuals or even across brain regions in a single individual, followed by limited transgene expression [3, 9]. Finally, inaccurate regional targeting can also occur, contributing to variability [10]. Here we report advances that help to address these limitations. First, to improve specificity of targeting, we chose to interfere with a mechanism that is specific for a distributed cell population, the striatal cholinergic interneurons. These neurons account for 1–2% of all striatal neurons [11]. We show for the first time in the old-world monkey that we can confine functional genetic targeting to cholinergic interneurons in the striatum by virus-mediated expression of shRNAs (RNAi) suppressing Choline Acetyltransferase (ChAT) [12]. In the same transcript we encode hM4Di_CFP, a chemogenetic receptor that can serve as a sensitive in vivo and in vitro reporter for monitoring gene expression [3, 10, 13]. Quantitative analyses by both visual classification and deep-learning assisted methods show that hM4Di_CFP expression and ChAT protein suppression are linearly correlated. hM4Di_CFP and shRNAs encoding RNAi are expressed in the same neurons. Using hM4Di_as a reporter allows to characterize the extent of the target region with the potential option of silencing activity in all expressing neurons [14], not just the ChAT rich neurons. Our approach should generalize to in vivo targeting, monitoring and verification of any genetic tool requiring expression of small RNAs in the primate brain, including for gene therapy applications in humans.

## Results

### Construct design and locations of striatum expression

**Fig. 1:**
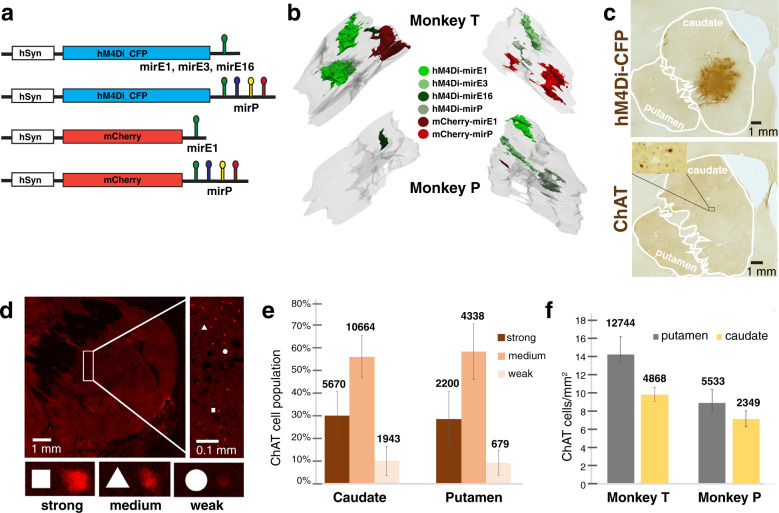
Categorization of ChAT expressing cells in the striatum following viral targeting. **a** Schematics of Lentivirus constructs. **b** Still image of 3D reconstruction of reporter expression using histology against the CFP or mCherry protein. Regions of at least 50% of neurons expressing the reporter were outlined on confocal images and included in the reconstruction of each injection. **c** Histochemistry against CFP (top) and the ChAT protein (bottom) on neighboring brain sections. Caudate and Putamen regions of the striatum were outlined to delineate regions of interest. **d** Visual categorization of ChAT expressing cells. Each cell positive for ChAT signal was categorized with markers (white square or triangle or circle) for either strong, medium or weak signal. **e** Strong, medium, and weak cells were counted (numbers on top of bars) and percentages plotted for the caudate and putamen respectively. **f** ChAT labeled cells in caudate and putamen (numbers on top of bars) were plotted as ratio of area in each section for either monkey. Error bars represent standard deviation in all graphs.

To express short hairpin RNAs (shRNAs) against ChAT mRNA along with a reporter, we designed three mirE-based monocistronic shRNAmirs [15] (Supplementary Fig. 1a). shRNAmir allows shRNAs to be placed into the context of a microRNA scaffold within the transcript of a messenger RNA while being correctly processed by the endogenous cell machinery [16]. As an alternative way of interferring with ChAT mRNA, we also used the scaffold of a polycistronic shRNAmir (Supplementary Fig. 1b) that was previously used successfully to inhibit HIV-1 replication in vitro [17]. Anti-ChAT siRNA candidates, shRNA sequences complementary to the ChAT coding sequence, were identified and ranked using the DSIR webtool (http://biodev.extra.cea.fr/DSIR/DSIR.html). The resulting siRNA oligonucleotides were then filtered further according to the following criteria: (a) they should be complementary to all known ChAT transcript isoforms [16] and (b) they should not have more than 15 nt complementarity to other sequences in the rhesus monkey transcriptome [18]. Of the remaining siRNA sequences, three were selected for the mirE scaffold, and four for the mir17-19b scaffold. The mirE-based shRNAmirs (mirE1, mirE3, and mirE16) and the polycistronic shRNAmir (mirP) were cloned into the 3′ UTR of a Lenti-hSyn::hM4Di_CFP construct [3]. The construct contained a fragment of the human synapsin promoter (hSyn) to make expression specific to neurons. mirE1 and mirP scaffolds were also cloned into a Lenti-hSyn::mCherry construct (Fig. 1a). Lentivirus constructs were injected via 10 µl infusion each into various sites distributed throughout the striatum of two monkeys. Expression regions located in either caudate or putamen were reconstructed by outlining contiguous reporter protein expression on sections within the regions of interest (Fig. 1b). A three-dimensional reconstruction of the injections sites is available at: 10.35092/yhjc.12616832. For expression analysis, regions of interest were outlined on each section, either putamen or caudate regions, depending on location of the injection (Fig. 1c). Volumes of expression regions varied widely from 1.5 to 15 mm^3^ (see Supplementary Table 1 for area outlined used for reconstruction) but penetrance, as judged by percentage of ChAT cells withing the injected regions also expressing reporter protein, was consistent, ranging from 72 to 84%, with most regions showing around 80% of ChAT positive cells also expressed the reporter protein (see Supplementary Table 1 for raw numbers).

### Categorization of ChAT expressing striatum neurons

Following antibody staining for ChAT protein and confocal imaging, we categorized all ChAT cells in the caudate or putamen of stained sections, based on fluorescence intensity as expressing either *strong*, *medium*, or *weak* levels of ChAT protein (Fig. 1d), without visualizing (blind to) reporter expression. Across 75 striatal regions outlined on sections, ratios of ChAT expression levels were similar in caudate and putamen of both monkeys, with ~30% of cells categorized as *strong*, 60% as *medium*, and 10% with *weak* expression of ChAT protein (Fig. 1e). The mean density of ChAT cells per unit area was 8–15 cells/mm^2^ (Fig. 1f). Raw cell counts are available in Supplementary Table 1.

**Fig. 2:**
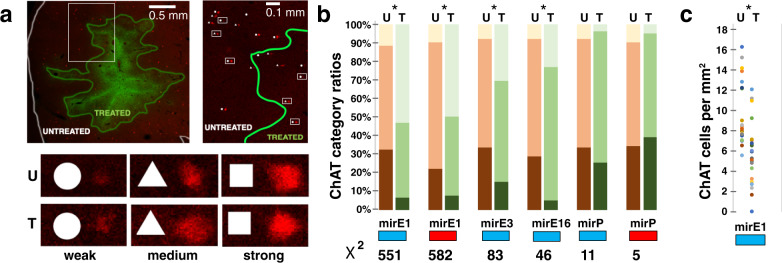
Categorization of ChAT expressing cells in treated and untreated regions. **a** Treated regions were outlined on sections double stained for ChAT (red) and reporter (green), with treated regions containing at least 50% of neurons expressing reporter. Categorized ChAT cells were additionally tagged for location in treated (T) or untreated (U) region. **b** Bar graph for categorization results. Blue rectangles for hM4Di_CFP reporter, red rectangles for mCherry reporter. Dark color in the bar plot denotes strong ChAT, medium color denotes medium ChAT, and light color denotes weak ChAT categorization as percentage of total. *X*^2^ Chi Square number. Asterisk (*) indicates significant with *p* < 0.001 (Chi square test). **c** ChAT cell counts per treated (T) or untreated (U) region plotted for each section containing staining for the hM4Di_CFP-MirE1 construct. *p < 0.001 (Wilcoxon–Mann–Whitney test). See Supplementary Table 1 for raw data.

To identify regions *treated* with a specific vector construct, we outlined clusters of cells with reporter expression, stained for CFP or mCherry, depending on the construct (Fig. 2a). We then plotted ratios of *strong*, *medium*, and *weak* ChAT expressing cells for *untreated* versus *treated* regions for each construct (Fig. 2b). As expected, *untreated* ratios were consistent with the overall striatal distribution for all constructs. In *treated* regions, there was a significant shift of these ratios towards weaker expressing cells for all four constructs with mirE scaffolds (Chi-square, *p* < 0.001, df = 2, see Fig. 2b for *X*^2^ values) but not for constructs with the polycistronic mir17-19b configuration (mirP, Chi-square, *p* > 0.1, df = 2, see Fig. 2b for *X*^2^ values). Both, the hM4Di_CFP-mirE1 and the mCherry-mirE1 construct showed the strongest ratio shift with <10% of cells being categorized as *strong*, 40% as *medium*, and 50% as *weak* ChAT expressing cells. The ChAT cell density for the hM4Di_CFP-mirE1 construct was reduced from 9.3 cells per mm^2^ to 5.6 ChAT cells per mm^2^ (approximately a 40% reduction, Fig. 2c, Wilcoxon–Mann–Whitney test *p* < 0.001). For other constructs cell densities were indistinguishable from the controls.

### ChAT and reporter expression are inversely correlated

**Fig. 3:**
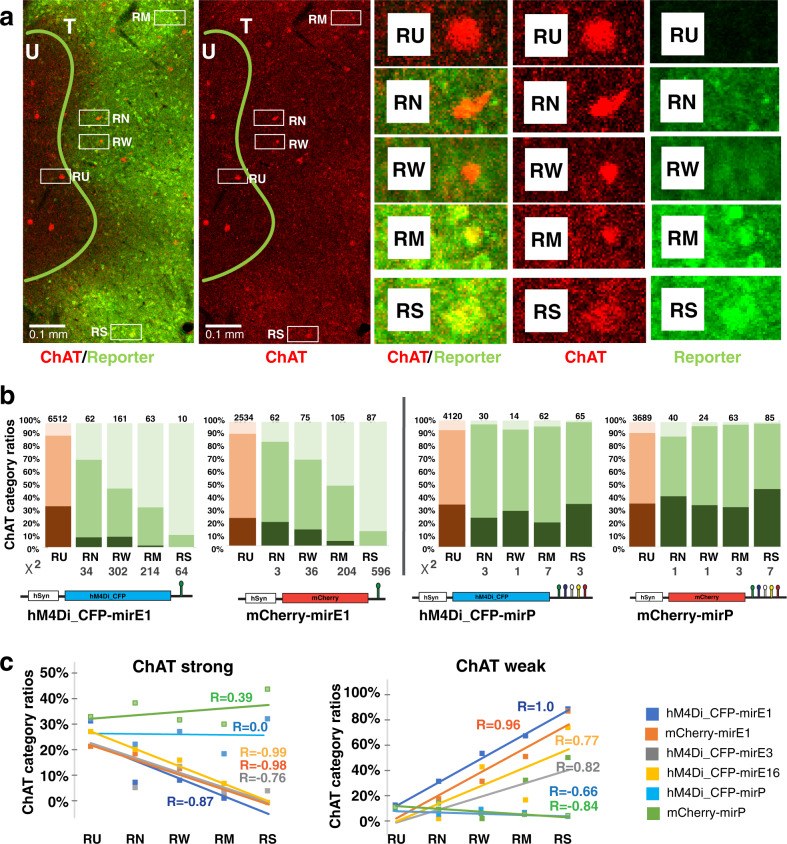
Correlation of ChAT expression ratios with reporter expression levels. **a** ChAT categorized cells were categorized for co-staining with reporter expression. RU untreated, RN reporter not detected (treated), RW weak reporter (treated), RM medium reporter (treated), and RS strong reporter (treated). **b** Percentages for ChAT categorized cells plotted for each reporter category. Distributions were compared to distribution of ChAT categories in surrounding untreated regions (RU). *X*^2^ Chi Square number. Asterisk (*) indicates significant with *p* < 0.001 (Chi square test). **c** Percentages of strong, medium, and weak ChAT categories plotted in relation to reporter categories (see Supplementary Table 1 for raw data).

Next, within the treatment boundaries we categorized the level of reporter expression (CFP or mCherry signal) for each ChAT expressing cell to: RN: “*reporter not detected*”, RW: “*weak reporter*”, RM: “*medium reporter*” or RS: “*strong reporter*”. ChAT cells in untreated regions were categorized as RU: “*untreated*” (Fig. 3a). When percentages of ChAT expression ratios were plotted in order from *untreated* (RU) to *strong reporter* expression (RS), the two mirE1 constructs showed a corresponding significant (Chi-square, *p* < 0.001) decrease in *strong* ChAT cell ratio and an increase in *weak* ChAT cell ratio (Fig. 3b, left panel). No cells that strongly expressed the mirE1 with either the hM4Di_CFP DREADD or the mCherry reporter were categorized as also strongly expressing ChAT protein. Increasing reporter levels and decreasing ChAT expression categories for these constructs were linearly inversely related (asymptotic linear by linear association test hM4Di_C: FP-mirE1: *Z* = 18.13, df = 8, *p* < 10^–16^, mCherry-mirE: *Z* = 18.5, df = 8, *p* < 10^–16^). Cell populations that strongly expressed the mirP scaffold together with either reporter showed distributions similar to control populations (Fig. 3b, right panel), with a slight correlational shift for the mCherry construct towards a higher ratio of *strong* ChAT cells, potentially explained by autofluorescence or signal leakage (hM4Di_CFP-mirP: *Z* = 0.31, *p* = 0.75, mCherry-mirP: *Z* = −2.5, *p* = 0.011). To illustrate the relative effect of virus expression on ChAT downregulation for each construct, we plotted the *strong* and *weak* cell proportions ordered according to reporter expression (RU, RN, RW, RM, and RS) for each construct and quantified using linear regression (Fig. 3c). For all mirE constructs, *strong* ChAT cell populations proportionally decreased with increasing reporter expression levels, while *weak* cell populations proportionally increased. This pattern was most pronounced for both mirE1 constructs (Fig. 3c). The asymptotic linear by linear association test also showed a significant correlation between ChAT decrease and reporter increase for hM4Di-mirE3 (*Z* = 8.72, df = 8, *p* < 10^–16^), and hm4Di-mirE16 (*Z* = 8.03, df = 8, *p* < 10^−16^).

### Deep-learning assisted analysis confirms correlation between reporter and RNAi

**Fig. 4:**
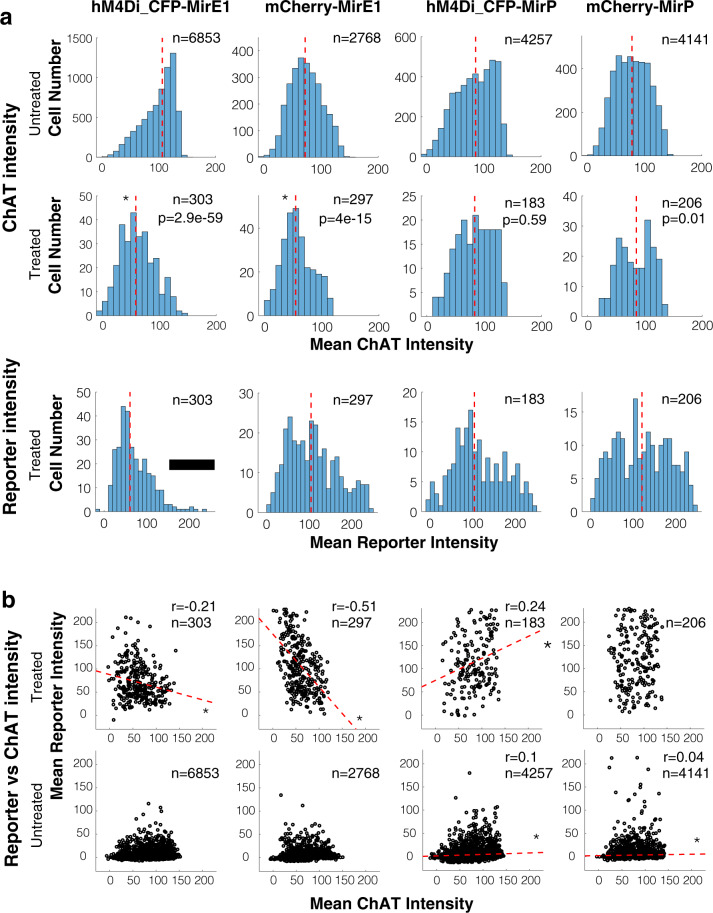
Expression analysis using deep-learning to outline ChAT expressing cells. **a** Histograms for ChAT cell numbers over intensity of ChAT or reporter signal (between 0 and 255). Red dashed line indicates median intensity of distribution. *p < 0.001 significant difference between median distribution in treated and untreated regions. Black bar: reduction in higher intensity reporter cells compared to other constructs. Kolmogorov–Smirnov test with Bonferroni correction. **b** Correlation plots between mean ChAT intensity and reporter intensity. Red dashed line indicates linear regression where significant using a Pearson correlation test.

Above, the ChAT staining intensity used for visual categorization was judged by eye. For an automated analysis, we trained a U-Net [19] deep-learning assisted imaging method (Supplementary Fig. 2) to measure the degree of ChAT and reporter staining in individual neurons by automatically outlining ChAT positive cells (Supplementary Fig. 3). For the U-net, one set of manually outlined ChAT positive neurons were used for training and a second manually outlined set of neurons were used for testing. The trained U-net was evaluated by how well the predicted segments by U-net matches the ground-truth segments labeled by human. There was a significant shift of the median distribution towards weaker ChAT signal in treated areas for both the mirE1 constructs (Fig. 4), as well as for the mirE3 construct (Supplementary Fig. 4a). Neither of the mirP constructs showed a significant change in median distribution (Fig. 4a). When reporter intensity was plotted against ChAT intensity, there was a significant inverse linear correlation for all mirE constructs, except mirE3 (Fig. 4b, Supplementary Fig. 4b). Additionally, there was a reduction of cells with high reporter in regions treated with hM4Di_CFP-mirE1 compared to other constructs, likely because ChAT signal suppressed by mirE1 in these cells was too weak to be detected by the outlining algorithm (black bar in Fig. 4a). As observed in the visual analysis, the deep-learning algorithm confirmed a weak but significant positive correlation for the hM4Di_CFP-mirP construct. Because the algorithm allowed correlation analysis in all regions, we were able to observe a similar positive correlation between reporter signal intensity and ChAT signal intensity in several untreated areas, without the presence of reporter (Fig. 4b). This small positive correlation is likely due to autofluorescence and/or a small leakage from the ChAT signal into the filter channel for the reporter signal.

### Acetylcholinesterase is not affected by ChAT shRNAmir

**Fig. 5:**
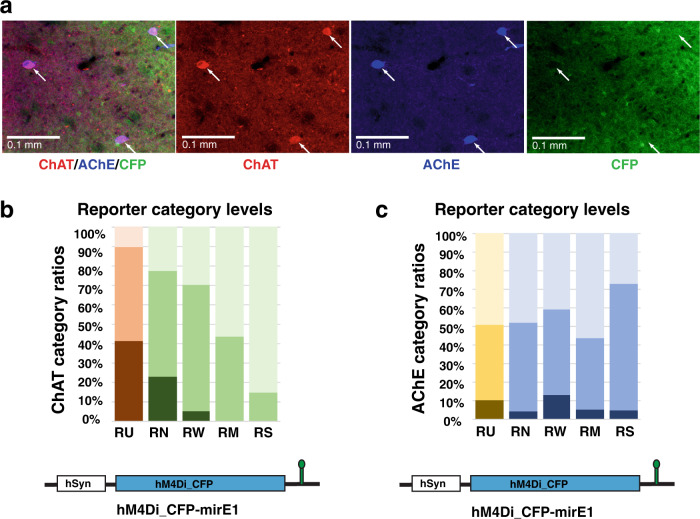
Correlation of ChAT expression ratios with CFP reporter expression levels for hM4Di_CFP-mirE treated regions. **a** Confocal multichannel image for colocalization of ChAT, AChE and hM4Di_CFP reporter protein. White arrows indicate the same cells in the four panels. **b** ChAT categorized cells (strong, medium, weak), categorized for co-staining with reporter expression—RU untreated, RN reporter not detected (treated), RW weak reporter (treated), RM medium reporter (treated), and RS strong reporter (treated). **c** AChE categorized cells (strong, medium, weak) were further categorized co-staining with reporter expression—labels as above. Distributions were compared to distribution of ChAT categories in surrounding untreated regions (RU). *X*^2^ Chi Square number. Asterisk (*) indicates significant with *p* < 0.001 (Chi square test). See Supplementary Table 2 for Raw Data.

To investigate whether shRNAmir mediated downregulation of ChAT protein would lead to changes in Acetylcholinesterase (AChE), an enzyme required for breakdown of Acetylcholine, we repeated the visual categorization on eight sections of striatal regions treated with hM4Di_CFP-mirE1 stained for Acetylcholinesterase (AChE) in addition to ChAT and CFP (Fig. 5a). As reported previously, in the striatum, both ChAT and AChE are expressed in the same neurons [20]. These sections were imaged with a ×10 objective, resulting in higher sensitivity but also thinner optical section, thus somewhat changing the relative distribution of ChAT expression categories in the untreated regions to 41% *strong*, 49% *medium*, and 10% *weak* (Fig. 5b). Analysis in treated regions again confirmed the strong inverse correlation of ChAT and CFP reporter protein ratios (Fig. 5b, *Z* = 13.13, df = 8, *p* < 10^−16^). On the other hand, there was no correlation between AChE and reporter protein ratios (Fig. 5c, *Z* = 0.48, df = 8, *p* = 0.62). Raw cell counts are available in Supplementary Table 1. These results strongly suggest that shRNAmir targets the ChAT production specifically, and at these levels, the ChAT decrease does not affect AChE expression.

## Discussion

The results above show for the first time in the non-human primate brain that we can use virally delivered shRNAmir to suppress ChAT protein expression in striatal interneurons thus functionally modifying a distributed neuron population. We can with the same transcript, from the same neuron specific promoter, express a protein as a reporter, here a chemogenetically inducible receptor. This combination of protein and shRNAmir expression has several advantages that are particularly important for using genetic tools in primates: first, by histological co-staining for reporter and ChAT protein, we show that we reduce the ChAT protein in correlation with the strength of shRNAmir expression. We further show that this reduction is specific to ChAT and does not appear to affect AChE, a protein in the cholinergic signaling pathway that has consistently been shown to covary with ChAT when the neurons have been targeted by chemical means [21]. Lentivirus mediated RNAi against ChAT has been successfully used before to target cholinergic neurons in the rodent septum [12], but in that case a GFP reporter was expressed from a separate promoter, so the ChAT suppression and promoter expression would likely not be tightly correlated. Recently, also in rodents, Qian et al. [14] used shRNAmir combined with hM4Di expression from lentivirus but did not quantify correlation between the two. ChAT activity is thought to be an essential step for cholinergic signaling [22] and in at least some of the neurons with strong reporter expression, ChAT protein levels were below detection (Figs. 2c, 4a). Our present study does not map the relationship between different levels of ChAT reduction and the degree of cholinergic signaling suppression.

Second, by co-expressing the shRNAmir and hM4Di_CFP from the same cell-type specific promoter, it will be possible to monitor expression in vivo via Positron Emission Tomography (PET) imaging [10, 13], potentially monitoring on and off cycles of dose-dependent Doxycycline gating [23]. PET imaging allows quantitative measurements of hM4Di expression, thereby allowing estimates of functional RNA interference levels, a feature invaluable for future human gene therapy applications [13]. Third, inducible silencing of the virus targeted region with the hM4Di_CFP receptor will be with the exactly the same subset of neurons that also express the shRNAmir. The behavioral effect of region-specific silencing via the hM4Di receptor would serve as a positive control for comparison to silencing of ChAT alone. In the current study, the regions for the individual virus injections were too small to expect behavioral effects from hM4Di_CFP silencing, but we previously established functional chemogenetic silencing for this specific receptor [3]. The usefulness of such a control was also displayed by Qian et al. [14] who used the combination of shRNAmir and hM4Di to convert glial precursor cells to dopaminergic neurons in the mouse brain; and then showed that these neurons are functional by reversing behavioral improvements when they chemogenetically silenced the converted neurons though activation of hM4Di. To our surprise, only constructs with mirE scaffolds resulted in successful ChAT suppression, while the polycistronic mirP constructs did not show any effect on ChAT expression. It would have optimal to have included scrambled shRNA constructs from the outset. This was not feasible because of the already large number of constructs. Thus, we decided to focus on the most promising constructs, using the untreated region as a control. We believe the fact that the polycistronic construct did not have an effect on ChAT expression provides an unintended but strong case that 3′ microRNA scaffolds in the context of reporter protein expression does not interfere with native gene expression.

Finally, the strategy is a proof-of-principle experiment, making it likely that this can be expanded to other targets of RNAi and CRISPR technologies requiring expression of small gRNAs [24], including those currently in development for treatment in human disorders. Due to the linear relationship between reporter and functional RNA interference, the strategy would be especially useful in applications where rather than a complete shutdown of a neuronal population, a moderate modulation of signaling output (monitored via PET) is desired [13]. Regional modulation of Dopaminergic or Serotonergic signaling pathways might be prime candidates of such applications. A major advantage of expressing shRNAmir from the 3′ untranslated region of an mRNA is the availability of cell-specific promoters (e.g., hSyn used in this study) and drug-gated transcription systems (e.g., via Doxycycline).

In summary, we present for the first time in old-world monkey brains that small RNAs can be expressed from the same transcript as a reporter protein, resulting in linear co-regulation of functional RNAi, and reporter expression. The technology presented here has the potential to modulate neurotransmitter signaling of a specific cell type during gene therapy, while monitoring strength of the effect in vivo via PET imaging.

## Materials and methods

### Construct design and cloning

All micro RNA scaffolds and RNAi sequences were designed in silico, using the Snap Gene Software (https://www.snapgene.com).

#### mirE constructs

The Designer of Small Interfering RNA algorithm (DSIR) was used to select 21nt long interfering RNA sequences (iRNA) targeting the rhesus ChAT mRNA. The DSIR algorithm produces iRNA that have two-nucleotide overhangs on both ends of the dsRNA. To create fully overlapping 22nt iRNA, the sense strand was modified by subtracting a nucleotide from the 3′ end and adding two bases to the 5′ end. The antisense sequence was modified by adding an overlapping nucleotide to the 5′ end. To create the mismatch corresponding to the endogenous mir-30A, the first nucleotide of the sense strand was changed from C/G->A or A/T->C. The 22nt sequences were then screened against the rhesus genome to eliminate sequences with potential off-target effects. Next, sequences were aligned with four isoforms of the ChAT mRNA. Sequences that did not align with all isoforms were disregarded. Finally, the three iRNA pairs that ranked highest (Supplementary Fig. 1a) were incorporated into the mir-E scaffold.

#### mirP constructs

The polycistronic anti-ChAT iRNA sequences were identified by the DSIR algorithm (Supplementary Fig. 1b). The same elimination steps discussed above were followed and the four most highly ranked sequences were selected and modified to mimic the endogenous mir17-19b [17]. Complete Scaffolds flanked by overlapping sequences of the pLenti-syn::hM4Di-CFP plasmid [3], including restriction site *BsRGI* and *AscI* flanking the 5′ and *SalI* flanking the 3′ end to offer the option of either restriction or In-Fusion cloning (Clontech). The DNA was synthesized and cloned into a pUC57 vector by Genewiz (https://www.genewiz.com). In-Fusion cloning (Clontech) was used to place scaffolds 3′ of the hM4Di-CFP open-reading frame of the pLenti-syn::hM4Di-CFP plasmid, digested with *AscI* and *SalI*. MirE1 and MirP constructs were also cloned into the pLenti-syn::mCherry plasmid in which the hM4Di-CFP open-reading frame was replaced by the mCherry open-reading frame via gene synthesis (Genwiz) and in fusion cloning. BrGI was used instead of AscI for digesting the plasmid before Infusion cloning (Clontech).

### Lentivirus production

Lentivirus production was performed as described in [4]. Third generation lentiviral vectors were produced by the co-transfection of 293T cells with pRRLsin backbone construct, helper plasmid (Pax2), pMD2.G, and pAdvantage (Addgene, Cambridge, MA, USA). New medium with Ultraculture (Invitrogen) was used to replace the supernatant after 24 h. The supernatant was collected after 24 h and subjected to ultracentrifugation at 22,000 rpm (Beckman S28 rotor) with a 20% sucrose cushion in PBS. The resulting pellets were resuspended in PBS, aliquoted and stored at −80 °C. The viruses were titered by transfecting 293T using serial dilutions of each viral aliquot and genomic DNA was obtained. A 150 bp lentivirus fragment was amplified and compared to a similar fragment from the endogenous human vasopressin receptor gene (hVAR1) using quantitative PCR.

### Surgery and virus injections

Two rhesus monkeys were used, one male (Monkey P) and one female (Monkey T) weighing 15 and 12 kg respectively. Experimental procedures followed the Institute of Laboratory Animal Research Guide for the Care and Use of Laboratory Animals and were approved by the National Institute of Mental Health Animal Care and Use Committee. Before any surgical procedure, an initial 3.0 T MR image with the animal in the stereotaxic frame was obtained to determine the location of the targeted area of the striatum. Surgical procedures were carried out using aseptic techniques under general anesthesia (induction via ketamine hydrochloride, 10 mg/kg, i.m.; maintained via isoflurane 1.0–3.0%, to effect) in a fully equipped operating room. Prophylactic antibiotics (Cefazolin, 15 mg/kg, i.m.) and immediate postsurgical analgesics (ketoprofen, 10–15 mg, i.m.), as well as a short course of ibuprofen, were administered. Injections were performed as described previously [25]. The stereotactic frame was custom made by Jerry Rig (NJ, USA). This custom stereotaxic frame is made of a lucite platform with non-magnetic aluminum arms and brass ear bars with vitamin E filled tips for visualization on the MR scan. An NIH Section on Instrumentation custom made tooth-marker mounted to a Kopf stereotaxic micromanipulator (kopfinstruments.com) was used as the fiducial system to ensure consistent placement of the animal within the stereotaxic frame [26]. Once in surgery, the ML and DV coordinates of the sagittal sinus were cross-referenced with the interaural zero coordinates at the center of the ear bars (AP, ML, and DV) for the stereotactic frame, to set the injection coordinates for each AP position. ImageJ (imagej.nih.gov) and Osirix (www.osirix-viewer.com) software was used to calculate the injection coordinates from the anatomical MRI scans. A Hamilton gastight syringe (www.hamiltoncompany.com, Reno, NV) affixed to a programmable infuse/withdraw syringe pump (https://www.harvardapparatus.com/remote-infuse-withdraw-pump-11-elite-nanomite-programmable-syringe-pump.html) mounted on a Kopf stereotaxic micromanipulator (kopfinstruments.com), with an NIH Section on Instrumentation custom made needle support foot, was used to perform the injections. For monkey P we injected the six different constructs in two different surgeries, 23 days apart. During the first surgery, three constructs were injected into the left striatum, three in the mid-caudate, and one in the mid-putamen (Fig. 1) at different AP levels calculated from the zero ear bars on the MRI. We have injected Lenti-hsyn::mCherry-mirE1 in the caudate at AP 31, Lenti-hsyn::hM4Di_CFP-mirP in the caudate at AP 26, and Lenti-syn::mCherry-mirP in the putamen at AP 27 as well as in the caudate at AP 23. During a second surgery, we injected six constructs into seven different loci at different AP levels: Lenti-syn::hM4Di_CFP-mirE1 was placed into the dorsal caudate of right striatum at AP 32, Lenti-syn::hM4Di_CFP-mirE3 was injected into two loci of the dorsal and ventral caudate of right striatum at AP 27 and at the same AP level, Lenti-syn::hM4Di_CFP-mirP was injected into the mid-putamen. Lenti-syn::mCherry-mirE1 and Lenti-syn::mCherry-mirP were injected in the mid-caudate and in the mid-putamen, respectively at AP 23. Lenti-syn::hM4Di_CFP-mirE16 was injected in the mid-caudate in the left striatum at AP 23. For monkey T, we injected the six constructs in ten different loci of left and right striatum at different AP level calculated form zero ear bars on the MRI scan. Lenti-syn::hM4Di_CFP-mirE1 was injected at AP 27 in the left mid-caudate and at AP 24 in the left dorsomedial putamen. Lenti-syn::hM4Di_CFP-mirE3 was injected at AP 27 in the right mid-caudate. Lenti-syn::hM4Di_CFP-mirE16 was injected at AP 24 in two loci of the dorsal and ventral caudate in the left striatum. Lenti-syn::hM4Di_CFP-mirP was injected at AP 24 in two loci of dorsal and ventral caudate in the right striatum. Lenti-syn::mCherry-mirE1 was injected at AP 20 in the left mid-caudate. Lenti-syn::mCherry-mirP was injected at AP 24 in the right mid-putamen and at AP 20 in the right mid-caudate. For both monkeys each construct was injected at rate of 1.0 µl/min for a total volume of 10 µl. To ensure adequate diffusion of the vectors, injection needles remained in place for a total of 10 min prior to a slow, staged withdrawal.

### Brain extraction and histology

After a minimum of 6 weeks of recovery, the animals were euthanized following AVMA guidelines. The animals were perfused transcardially with heparinized saline followed by a solution of 4% or paraformaldehyde in 0.1M phosphate buffer. For each case, the brain was removed and cryoprotected through an ascending series of glycerol solutions. The cryoprotected tissue was then frozen in isopentane and serially sectioned (at 40 µm) using a sledge microtome. Series with every tenth section (400 µm apart) of free-floating sections were processed for either histochemistry using the ABC Elite Kit (Vector laboratories PK-6100) or via immuno-visualization of ChAT, AChE, NeuN, and CFP or mCherry reporters. Primary Antibodies: anti-CFP rabbit polyclonal (Abcam ab290), goat polyclonal anti-ChAT (Millipore AB144P), rabbit polyclonal anti-mCherry (Abcam ab167453), goat anti-mouse Alexa 647 (Invitrogen A21235), mouse anti-AChE (Ivitrogen MA3-042). Secondary Antibodies: biotinylated anti-rabbit (Vector laboratories BA1000), goat anti-mouse Alexa 647 (Invitrogen A21235), goat anti-rabbit Alexa 555 (Invitrogen A21428), goat anti-chicken Alexa 488 (Invitrogen A11039), and goat anti-rabbit Alexa 488 (Invitrogen A11008).

### Confocal microscopy

Images of the striatum were collected by confocal microscopy using a Zeiss LSM780 laser scanning confocal microscope. The channels selected for visualizing the viral constructs, ChAT cells, and neural cells were 488, 647, and 555 nm, respectively. For each image, the laser power was adjusted accordingly in order to enhance visualization of the neurons. Images used for analysis were taken with a ×5 objective.

### Cell counting and 3D reconstruction

Following image collection, the sections were aligned and compiled into stacks using BrainMaker (MBF Bioscience), with 400 µm in between sections. A stack was created for each brain hemisphere of each monkey. The image stacks were then imported into Neurolucida360 (MBF Bioscience) for analysis. The striatal region of interest (caudate or putamen) was outlined using the Contour mode of the program. Area measurements of striatal regions and treated regions were calculated from these outlines by the program. While only visualizing the ChAT channel, markers were used to count and categorize the ChAT positive cells as either strong, medium, or weak. The injection region was similarly outlined using only the relevant channels. ChAT cells within the injection region were also counted and categorized as either having strong, medium, weak, or no viral expression. This process was repeated for all sections within the stacks. 3D reconstructions of each brain hemisphere were created using the 3D environment of Neurolucida360 to visualize the viral expression in the monkey striatum. Movies of the reconstructions are available at: 10.35092/yhjc.12616832.

### Deep-learning assisted analysis

ChAT positive cells were outlined automatically with U-net and ImageJ. First, images were subjected to segmentation. We adapted a pretrained U-net, which was added to ImageJ as a plugin [19], to outline ChAT positive cells for each monkey. Each training region (2000 × 2000 pixels) and test region (1000 × 1000 pixels) were randomly selected from different sections. For Monkey P, 443 neurons from four regions were manually outlined to train U-net and 149 neurons from four regions were used for testing. For Monkey T, 787 neurons from five regions were manually outlined for training and 117 neurons from five regions for testing. Both training and testing images were black–white inverted by ImageJ. The trained U-net was evaluated with test images by intersection over union (IoU), which measures how well the predicted segments by U-net matches the ground-truth segments labeled by human. Briefly, for each pair of predicted and ground-truth objects, IoU was calculated by dividing the intersection of these two objects by their union [19]. A value of 0 indicates no overlap and 1 indicates exact overlap. For both monkeys, average IoUs were above 0.7 (Supplementary Fig. 2), which indicates a good segmentation [19]. All the sections were then segmented by the trained U-net and individual ChAT positive neurons were outlined by ImageJ (Analyze-Analyze Particles). Each section was inspected carefully to remove false positive “cells” and pickup missed cells. To make ChAT fluorescence intensity comparable across different sections, background intensity of putamen or caudate was adjusted to the same level (about 100). Intensity indicates gray levels of the images or pixels, ranging from 0 to 255 for our 8-bit images. Background intensity was defined as the mean intensity of all pixels in putamen or caudate. For each ChAT positive cell, mean intensity was calculated as averaged intensity of all pixels in the cell body with background intensity subtracted. The area of a cell was defined as the pixel numbers occupied by the cell body. To analyze reporter signals within ChAT positive cells, we transferred all the outlines of ChAT positive cells to the reporter channel. Similarly, intensity of putamen or caudate in reporter channel was adjusted to normalize the background intensity (about 10). Background intensity was defined as the mean intensity of regions removed from the injection site. The mean intensity of reporter signals within ChAT cells were calculated in the same way as for the ChAT signal.

### Statistics

All cell counts and area measurement of each region for ChAT and Reporter categories displayed in Figs. 1–3 are listed in Supplementary Table 1. Cell counts for each region for ChAT or AChE and Reporter categories displayed in Fig. 5 are listed in Supplementary Table 2. Error bars in Fig. 1 represent Standard Deviation of category proportions or cells per area between regions. Chi square values in Figs. 2b, 3b, 5b, c were calculated with help of the R software package (https://www.r-project.org) by first determining the ChAT expression category ratio of all cells in untreated regions and then asking if the cell numbers distributed between the categories in treated regions for each construct resulted in a significantly different category ratio. The asymptotic linear by linear association test was then performed by asking whether the ChAT or AChE ratios were significantly ordered according to RU > RN > RW > RM > RS. R values in Fig. 3c was calculated using a Pearson’s correlation on either strong or weak ChAT ratios ordered according to RU > RN > RW > RM > RS.

The significance between treated and untreated regions in cells per area in Fig. 2c was established by determining the cell density for each region and then testing the densities for treated vs. untreated regions via a Wilcoxon–Mann–Whitney test. No Bonferroni correction was performed.

## Supplementary information

Reporter coregulated shRNAmir supplement
