# Supplementary material for: RNAi and chemogenetic reporter co-regulation in primate striatal interneurons
Source: Gene Ther. 2021 May 19;29(1-2):69–80. doi: 10.1038/s41434-021-00260-y (PMC8856958; doi:10.1038/s41434-021-00260-y)
Supplement: Supplementary file 1 — Reporter coregulated shRNAmir supplement [file 41434_2021_260_MOESM1_ESM.docx]

**RNAi and chemogenetic reporter coregulation in primate striatal interneurons**

Walter Lerchner^1^*, Abdullah A. Adil^1^, Sekinat Mumuney^1^, Wenliang Wang^1^, Rossella Falcone^1^, Janita Turchi^1^ and Barry J. Richmond^1^*
^1^*Laboratory of Neuropsychology, NIMH, NIH, Bethesda, United States*

**Supplementary Information**

**
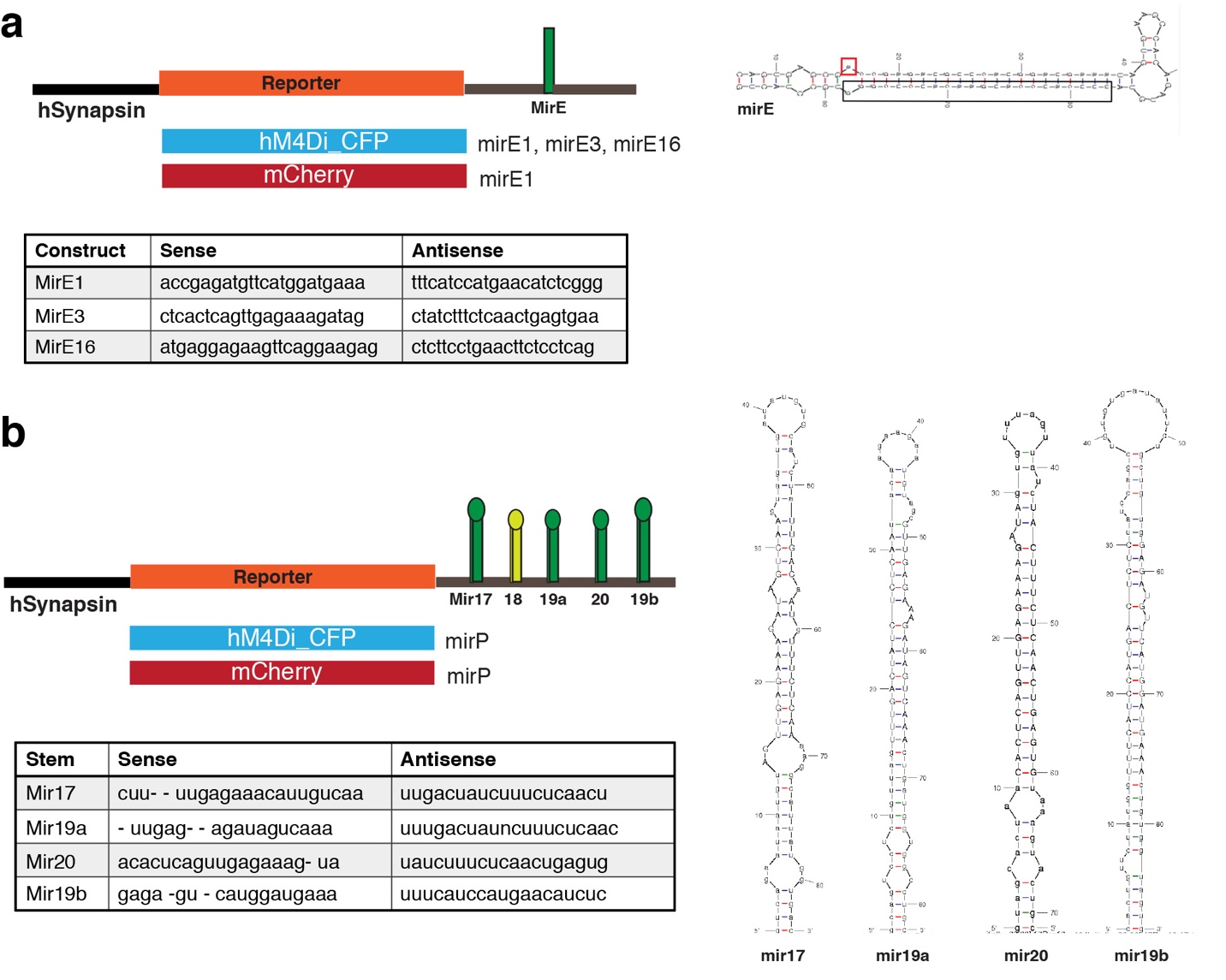
**

**Supplementary Figure 1.** Construct design. (**a**) MirE constructs. ChAT specific sequences were inserted into a MirE scaffold to result in MirE1, MirE3 and MirE16 constructs (**b**) MirP constructs. ChAT specific sequences were inserted into a single Mir17-19b scaffold as described by Liu et al.^17^


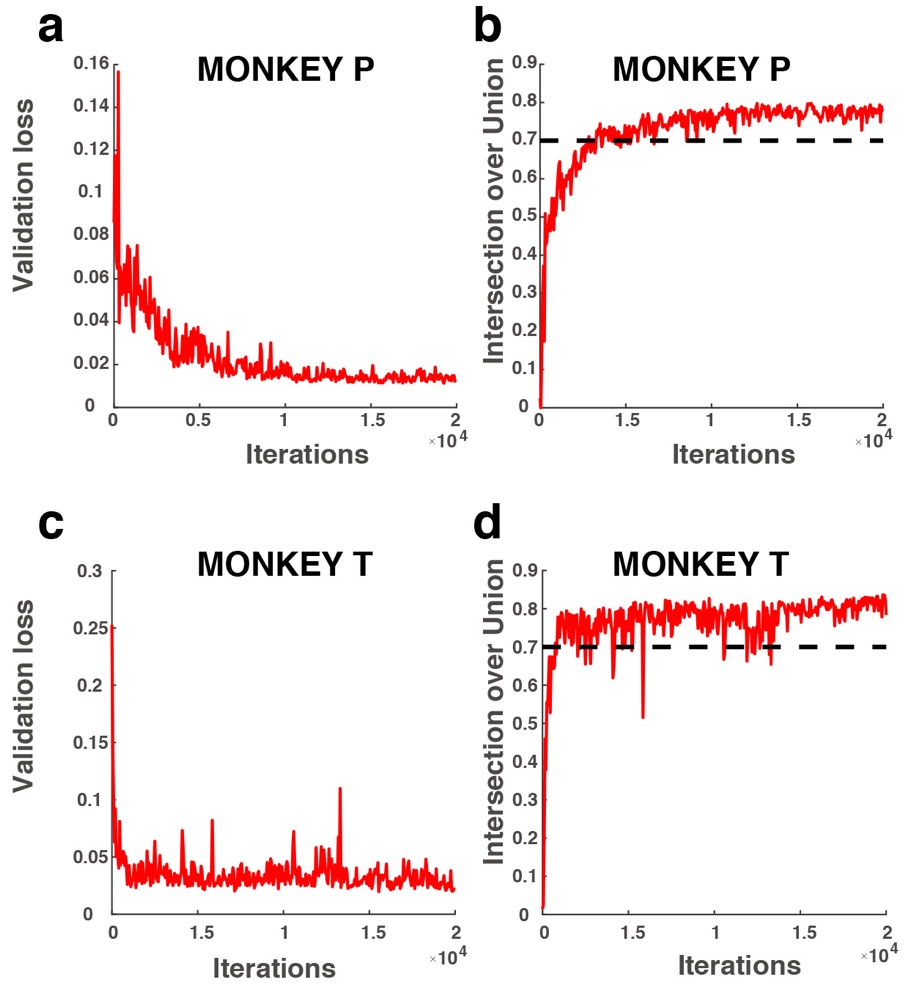


**Supplementary Figure 2.** Evaluation of U-net transfer learning during training. (**a, b**) Segmentation performance measured as validation loss (a) and IoU (b) during training for Monkey P. (**c,** **d**) Same as a,b except for Monkey T. Input patch size: 380 × 380, solver: ADAM, base learning rates: 5 x 10^-6^ (fixed schedule), momentum: 0.9, momentum2: 0.999, iterations: 20000.


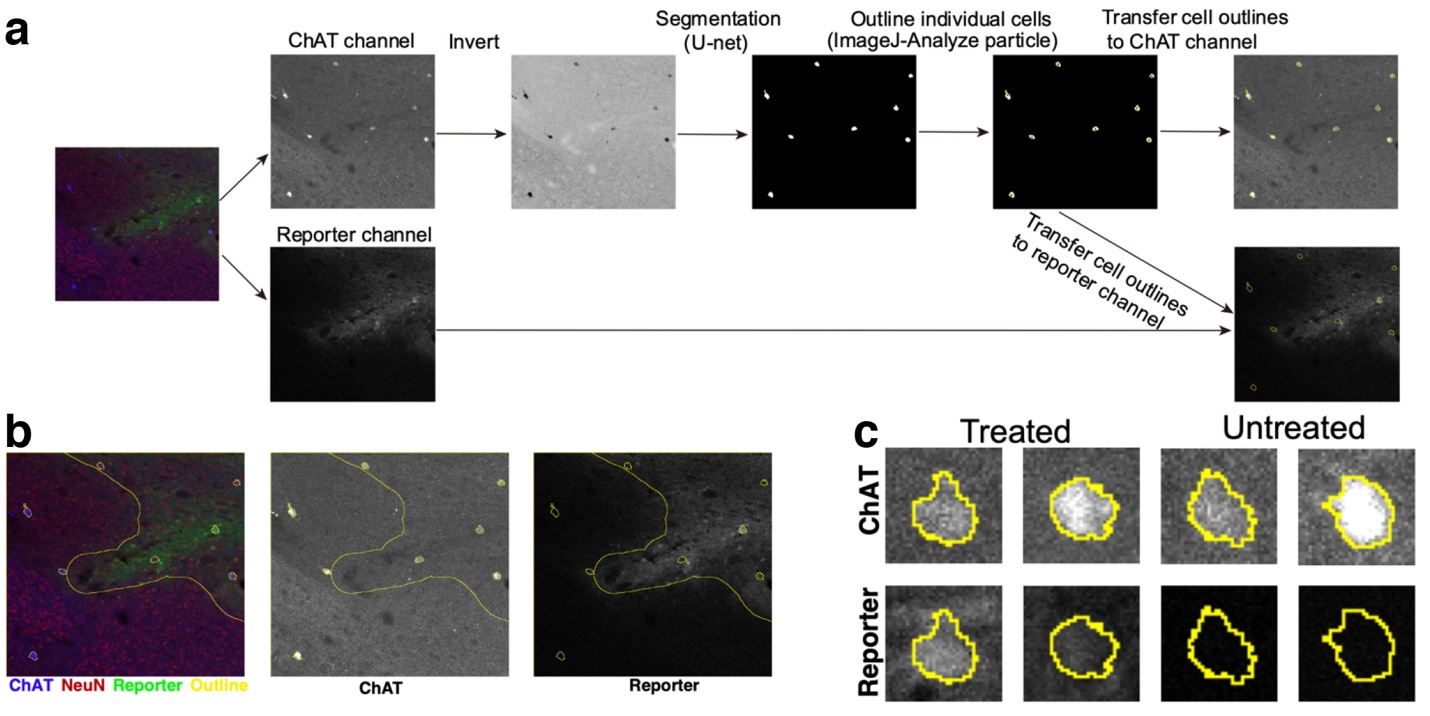


**Supplementary Figure 3.** ChAT and reporter intensity analysis by deep learning assisted outlining of ChAT expressing cells. (**a**) Workflow of cell outlining in ChAT and Reporter channels. (**b**) Outlining of treated region using reporter expression. (**c**) Deep learning assisted outlines of ChAT expressing cells in treated and untreated regions.


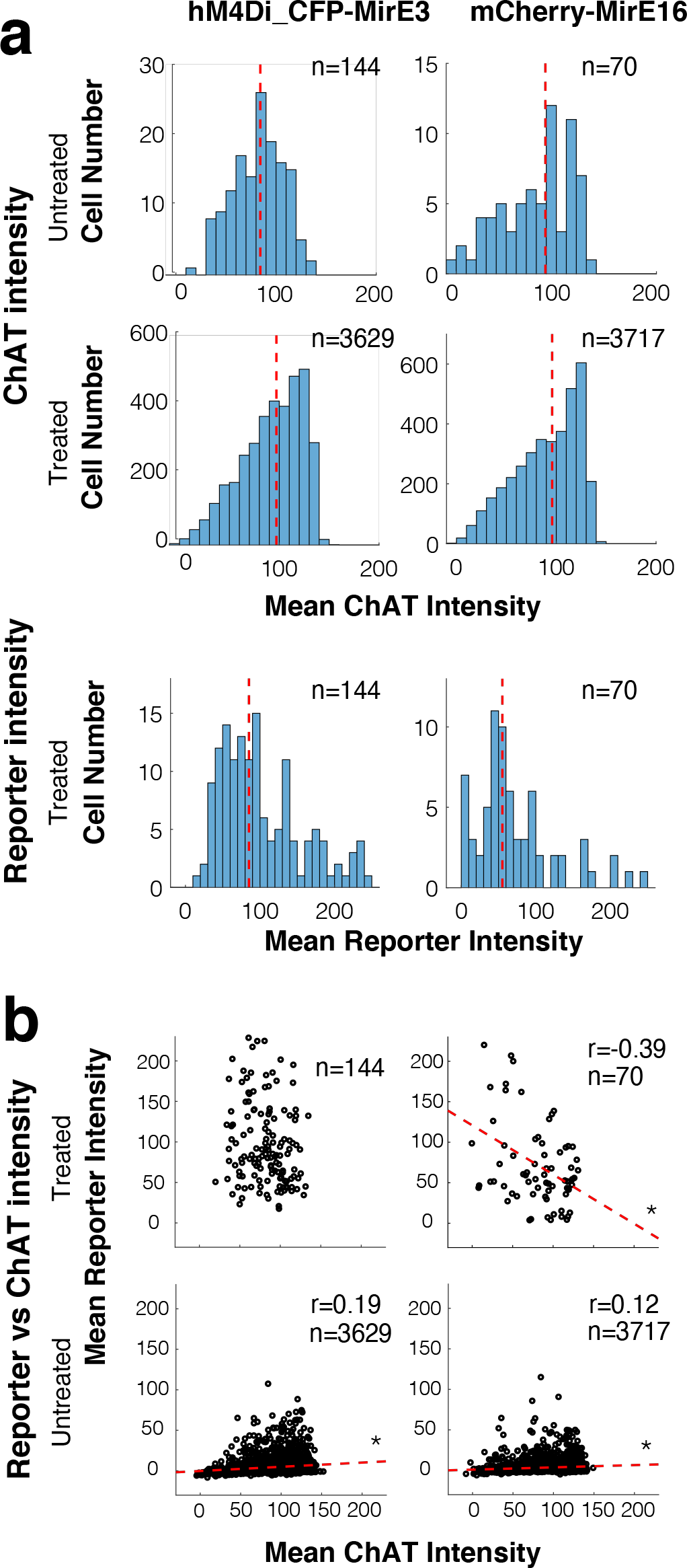


**Supplementary Figure 4**. Expression analysis using deep learning to outline ChAT expressing cells. (**a**) Histograms for ChAT cell numbers over intensity of ChAT or reporter signal (between 0 and 255). Red dashed line indicates median intensity of distribution. *: p < 0.001 significant difference between median distribution in treated and untreated regions. Kolmogorov-Smirnov test with Bonferroni correction. (**b**) Correlation plots between mean ChAT intensity and reporter intensity. Red dashed line indicates linear regression where significant. *: p < 0.001.

**Supplementary Table 1.** Cell counts and area measurement of each region for ChAT and Reporter categories displayed in Figs. 1-3.

**Supplementary Table 2.** Cell counts for each region for ChAT or AChE and Reporter categories displayed in Fig. 5.
